# Supplementary material for: Causal Relationship between Aging and Anorexia Nervosa: A White-Matter-Microstructure-Mediated Mendelian Randomization Analysis
Source: Biomedicines. 2024 Aug 16;12(8):1874. doi: 10.3390/biomedicines12081874 (PMC11351342; doi:10.3390/biomedicines12081874)
Supplement: Supplementary file 1 [file biomedicines-12-01874-s001.zip › Supplementary Figure/Figure S8/Leaveoneout plot of MR analysis for TL to ubm-b-1741.pdf]

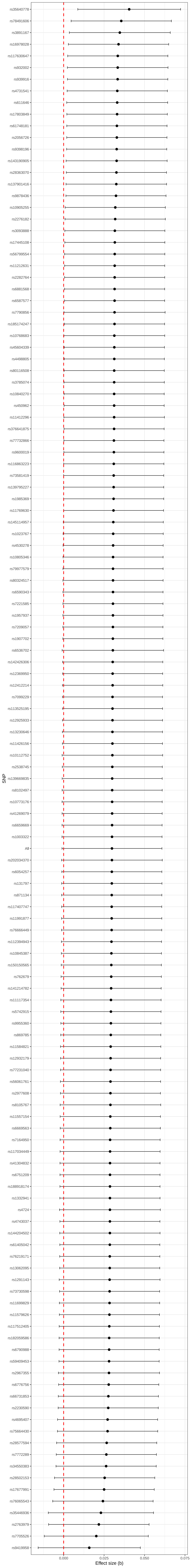

rs35640778
rs78491606
rs3891167
rs16978028
rs117630647
rs932002
rs939916
rs4731541
rs611646
rs17803849
rs61748181
rs2056726
rs9398196
rs143190905
rs28363070
rs137901416
rs9878436
rs10905255
rs2276182
rs3093888
rs17445108
rs56799554
rs11212631
rs2282764
rs6881568
rs6587577
rs7790856
rs185174247
rs10768683
rs45604339
rs4498805
rs80116508
rs3785074
rs10840270
rs450962
rs11412296
rs376641875
rs77732868
rs9600019
rs116863223
rs73581419
rs139795227
rs1985369
rs11769630
rs145114957
rs1023767
rs4530278
rs10805346
rs79977579
rs80324517
rs6590343
rs7221585
rs1957937
rs7209057
rs1907702
rs6536702
rs142426306
rs12369950
rs12412214
rs7099229
rs113525195
rs12925933
rs13230646
rs11426156
rs10112752
rs2538745
rs139669835
rs8102497
rs10773176
rs41269079
rs6659669
rs1003322
All
rs202034370
rs6054257
rs131797
rs871134
rs117407747
rs11991877
rs76666449
rs112394943
rs10845387
rs150150565
rs762679
rs141214782
rs11117354
rs5742915
rs9955360
rs869785
rs11584821
rs12932179
rs77231040
rs56061761
rs2977608
rs8105767
rs11557154
rs6669563
rs7164950
rs117034449
rs41304832
rs6751209
rs188918174
rs1332941
rs4724
rs4743037
rs144204502
rs61405042
rs76219171
rs13062095
rs1291143
rs73730598
rs11699829
rs11579626
rs117512405
rs182059586
rs6790988
rs59409453
rs2967355
rs6776756
rs66731853
rs2230590
rs4695407
rs75664430
rs28577594
rs7772289
rs34550383
rs28502153
rs17677991
rs76065543
rs35446936
rs2763979
rs7705526
rs9419958
SNP
0.000
0.025
0.050
0.075
Effect size (b)
